# Individual and social determinants of COVID-19 vaccine uptake

**DOI:** 10.1186/s12889-021-10862-1

**Published:** 2021-04-28

**Authors:** K. Viswanath, Mesfin Bekalu, Dhriti Dhawan, Ramya Pinnamaneni, Jenna Lang, Rachel McLoud

**Affiliations:** 1 Harvard T. H. Chan School of Public Health and Dana-Farber Cancer Institute, Boston, Massachusetts USA; 2 Harvard University, Cambridge, Massachusetts USA; 3 Dana-Farber Cancer Institute, Boston, USA

**Keywords:** COVID-19 vaccine uptake, Social determinants and vaccine uptake, Partisanship and vaccine acceptance, Trust in scientists

## Abstract

**Background:**

COVID-19 has had a devastating impact and efforts are being made to speed up vaccinations. The growing problem of vaccine hesitancy may affect the uptake of COVID-19 vaccine. We examined the individual, communication and social determinants associated with vaccines uptake.

**Methods:**

Data come from a nationwide online probability-based panel of 1012 representative adults in the United States and the survey was conducted before the vaccines were available. People under the federal poverty level and racial and ethnic minorities were oversampled. Our outcome variables of interest were likelihood of vaccinating self and likelihood of vaccinating people under one’s care (such as children) measuring behavioral intentions. Independent variables included perceptions of risk, exposure to different media for COVID-19 news, political party identification, confidence in scientists and social determinants of health. Logistic regression analysis was used to ascertain the effects of independent variables on the two outcome variables.

**Results:**

The results indicated that 68 and 65% agreed to get the vaccine for themselves and people under their care, respectively. Risk perceptions (severity of and susceptibility to COVID-19) were significantly associated with vaccine uptake. People who relied on “conservative” news outlets, Republicans, and who had low confidence in scientists are least likely to vaccinate self or children. Non-Hispanic Blacks and those with least schooling were also less likely to receive vaccine for themselves or people in their care.

**Conclusions:**

Our study identified race/ethnicity, risk perceptions, exposure to different media for COVID-19 news, party identification and confidence in scientists as factors that would be affecting COVID-19 vaccine uptake. The good news is that these are addressable through strategic public health communications, but a lot of work remains to be done with some urgency.

## Background

The emergence of SARS-CoV-2 in late December in China and the consequential spread of the infection, COVID-19, across the globe has had devastating impact unmatched by any other infection in the recent times. Globally, the new Coronavirus has infected close to 132 million people with more than 2.8 million deaths as of April 7, 2021 [1]. In the United States alone, the number of COVID-19 cases surpassed 30.5 million with more than 552,000 deaths [1]. The infections and associated morbidity and mortality continue to increase worldwide with intermittent flareups even in countries that were assumed to have brought it under control. Prior to the introduction of the COVID-19 vaccine and effective experimental treatments, countries have been relying on a combination of non-pharmaceutical interventions (NPIs) such as face coverings and physical distancing, and policy measures such as severe restrictions on public gatherings, temporary closure of institutions and work from home (WFH) policies.

As a result of a concerted global effort, including Operation Warp Speed in the United States, about 8 vaccines are now in use across the globe. The development and deployment of vaccines with such great speed is impressive with almost another 50 candidates in play at various stages of development [2]. With the introduction of vaccines, and several promising candidate vaccines entering Phase 2 and Phase 3 clinical trials, discussion has turned to the issue of implementation and acceptance of vaccine by the public. There are calls for more proactive efforts to promote COVID-19 vaccination [3].

The steep decline in vaccine preventable diseases (VPD) are one of the greatest public health success stories. Yet there is a small but significant group of people who have refused to get their children vaccinated and are vehemently opposed to childhood vaccines of any kind [4]. The anti-vaccine sentiment gets more attention than the actual number of vaccine refusers and appear to have disproportionate impact on public discourse. In turn, this persistent questioning of vaccine safety despite overwhelming scientific evidence has increased vaccine *hesitancy*, questions about safety and efficacy of vaccines among some parents even if they comply with the vaccination recommendations [5].

In addition to challenges with vaccine hesitancy on childhood immunization, the track record for adult vaccination, especially against influenza, is poor. For example, in the United States, despite repeated campaigns, the proportion of adults who get flu vaccines remains low: 45% [6].

Given this record of poor adult vaccination rates and growing vaccine hesitancy if not outright anti-vaccine sentiment against childhood immunization, the question is the extent to which people are open to COVID-19 vaccination given that they are now available and are an essential tool to stem the pandemic. In this paper, drawing on a nationally representative sample of American adults, we examined the individual, communication and social determinants that are associated with COVID-19 vaccines acceptance for themselves and for people under their care, for example, children.

## Methods

### Study design and study population

The data for this study come from a large nationwide online probability-based panel (KnowledgePanel®) in the United States. The KnowledgePanel was created by combining random digit dialing and address-based sampling methods. The combination of these two methods of creating the panel helped overcome the problems associated with cell phone penetration that affected many random digit dialing surveys. Because surveys among KnowledgePanel members are conducted online, panel members who did not have internet access were provided a web-enabled device and free internet service. Participants were eligible for the study if they were adult (18 years or older).

Data were gathered from 1012 nationally representative adults with an oversampling of those under the federal poverty level and racial and ethnic minorities, especially blacks and Hispanic groups. Our sample size was set a priori to achieve a margin of error of 4.1% and a confidence level of 95%. Assuming a response rate of 60%, based on American Association of Public Opinion Research (AAPOR) standards, 1822 panel members were contacted to reach our target sample size [7].

### Survey administration and data collection

The survey was administered in English and Spanish in July 2020. As part of KnowledgePanel’s standard procedure, the survey instrument was pretested among 25 participants. The selected respondents were sent an invitation to participate in the survey via email. Email reminders were sent to non-responders on day 3, day 6, day 9 and day 11 of the field period. Participants were given incentives such as special raffles and sweepstakes with both cash rewards and other prizes to be won.

### Measures

#### Outcome variables

The two outcome variables are likelihood of vaccinating self, and likelihood of vaccinating people under one’s care (such as children) representing behavioral intentions. These were measured by asking the question: “If a vaccine for COVID-19 becomes available during the next 12 months, how likely do you think you would get it for yourself” and “people under your care (e.g. children)” on a four-point scale: 1 = Not at all likely, 2 = Unlikely, 3 = Likely, 4 = Very likely. For analysis, we dichotomized the responses into “likely” and “unlikely”. The respondents were asked to answer separately for each outcome.

#### Independent variables

These included perceptions of risk, exposure to different media and platforms for COVID-19 news, political party identification, trust in science, and social determinants of health including education, income and race and ethnicity.

*Perceptions of risk* had two dimensions: perception of susceptibility and perception of severity, and each was measured using three questions on a four-point scale. Using the sum scores, we computed a high vs. low susceptibility and severity variables. We also measured if anyone in their family has been diagnosed with COVID-19 to assess perceptions of risk.

*Exposure to different media and platforms for COVID-19 news* was measured by providing respondents with six groups of media and platforms adapted from previous research [8]. The six groups included (1) mainstream print outlets (such as the Associated Press, *The New York Times*, the *Washington Post*, or the *Wall Street Journal*), (2) conservative outlets (such as Fox News, Rush Limbaugh, Breitbart News, One America News, or The Drudge Report), (3) liberal sources (such as MSNBC, Bill Maher, or Huffington Post), (4) mainstream broadcast (such as ABC News, CBS News, or NBC News), (5) online media news aggregators (such as Google News or Yahoo News), and (6) social media sources (such as Facebook, Twitter, or YouTube) [8]. The basis of this classification of ideology is supported by a recent study by the Pew Research Center [9]. We asked the respondents to indicate whether each of them is their “major source”, “minor source” or “not a source” for COVID-19 information. For analysis, we dichotomized the responses into “major source” and “not a major source”.

*Political party identification* was measured by asking respondents to identify themselves as republican, democrat, independent or other.

*Confidence in Scientists* was measured by asking respondents to indicate whether they have “a great deal of confidence”, “only some confidence” or “hardly any confidence at all” in scientists or researchers working on science. For analysis, we dichotomized the responses into “a great deal of confidence” and “hardly any confidence”.

We used standard and commonly used questions to measure social determinants of health including, gender, age, education, income, employment, and race/ethnicity.

### Statistical analysis

Frequencies of our independent variables across the two outcome variables were obtained to describe the study sample. We then built logistic regression models to ascertain the effects of perceptions of risk, exposure to different media and platforms for COVID-19 news, political party identification, confidence in scientists, and the social determinants of health on our two outcomes – likelihood of vaccinating self and likelihood of vaccinating people under one’s care (such as children). Each predictor was tested in a separate model controlling for age, gender, race/ethnicity, education, and income. Odds ratios and corresponding 95% confidence intervals were calculated. Because the 95% confidence intervals have not been adjusted for multiple testing, the associations should not be used to infer definitive effects. All statistical analyses were performed with R version 3.6.1 (2019-07-05). The analyses were conducted in September 2020.

## Results

In our sample, about 68% said that they were ready to get the vaccine for themselves and 65% agreed that they would vaccinate people under their care such as children. In general, the proportion of sample who expressed the likelihood of getting the vaccine themselves is higher than the likelihood of vaccinating people they cared about, for example children (Table 1).

**Table 1:** Weighted frequencies of demographic characteristics and independent variables across the two outcome variables

Predictors	Likelihood of vaccination of Self (*N* = 992) (%)*	Likelihood of vaccinating people under their care (*N* = 964) (%)*
Total, *N* = 1012 (%)*	68.3%	65.1%
Risk Perceptions		
Susceptibility		
Unlikely (50.3%)	57.9%	55.2%
Likely (49.7%)	78.3%	74.8%
Severity		
Not so serious (64.0%)	62.6%	57.4%
Serious (36.0%)	79.5%	79.6%
Personal Experience		
No (84.9%)	74.5%	73.9%
Yes (15.1%)	56.5%	66.7%
News Sources		
Conservative Outlets		
Not a major source (82.4%)	70.4%	68.0%
Major Source (17.6%)	58.4%	51.1%
Liberal Sources		
Not a major source (88.6%)	68.3%	65.4%
Major Source (11.4%)	71.1%	66.0%
Mainstream Broadcast		
Not a major source (83.8%)	72.1%	69.2%
Major Source (16.2%)	60.5%	60.0%
Mainstream Print		
Not a major source (71.6%)	62.4%	58.8%
Major Source (28.4%)	83.3%	81.2%
Online Media		
Not a major source (83.6%)	68.4%	64.1%
Major Source (16.4%)	72.4%	74.8%
Social Media		
Not a major source (90.5%)	68.7%	65.0%
Major Source (9.5%)	66.0%	68.1%
Ideologies		
Political party identification		
Independent (31.6%)	68.9%	66.2%
Republican (28.8%)	58.5%	53.5%
Democrat (39.5%)	76.1%	73.2%
Confidence in scientists		
Hardly any confidence (42.2%)	51.6%	47.5%
A great deal of confidence (57.8%)	80.9%	78.1%
Social and Demographic Characteristics		
Age		
18–24 years (20.8%)	73.0%	67.2%
30–44 years (25.0%)	56.8%	59.5%
45–59 years (24.8%)	72.2%	70.7%
60+ years (29.4%)	71.5%	63.7%
Gender		
Male (48.5%)	68.9%	64.5%
Female (51.5%)	67.7%	65.7%
Income		
< $10,000 (3.3%)	63.5%	62.1%
$10,000 to <$25,000 (10.3%)	52.6%	44.0%
$25,000 to <$50,000 (18.2%)	65.0%	62.1%
$50,000 to <$75,000 (17.0%)	68.6%	70.5%
$75,000 to <$100,000 (13.8%)	70.1%	65.7%
$100,000 to <$150,000 (17.7%)	71.4%	66.7%
> $150,000 (19.6%)	76.0%	72.7%
Education level		
Less than high school (10.7%)	63.8%	55.0%
High school (28.4%)	58.9%	56.8%
Some college (27.8%)	65.5%	59.6%
Bachelor’s degree or higher (33.2%)	79.6%	79.3%
Race/Ethnicity		
White, Non-Hispanic (63.2%)	71.8%	68.0%
Black, Non-Hispanic (11.8%)	51.0%	53.0%
Hispanic (16.5%)	64.9%	63.0%
Other, Non-Hispanic (8.5%)	72.4%	64.2%
Employment Status		
Employed (59.9%)	63.9%	62.3%
Laid off/Furloughed (5.7%)	64.4%	70.2%
Retired (23.1%)	74.1%	66.1%
Homemaker (6.2%)	71.9%	67.9%
Full-time student (5.1%)	96.9%	95.2%

* Indicates weighted percentages of participants with that particular characteristic

We then examined the association of the two outcomes with the different individual and social factors: perceptions of risk [10], exposure to different media and platforms for COVID-19 news, political party identification, confidence in scientists, and social determinants of health.

### Perceptions of risk and vaccine uptake

**Fig. 1:**
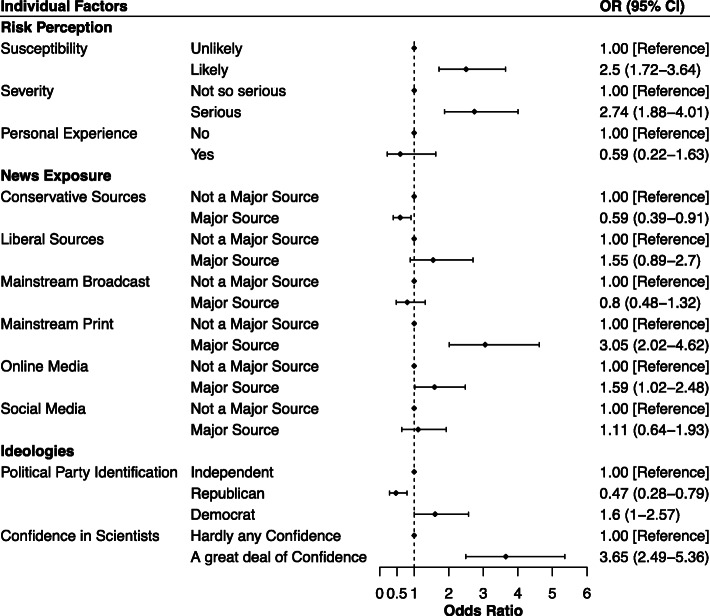
Odds ratios and 95% CI plots for predictors of likelihood of vaccinating self

**Fig. 2:**
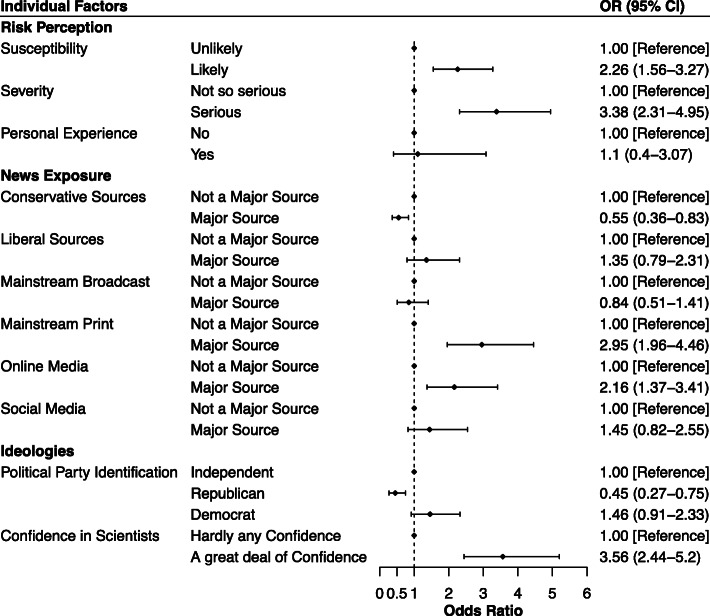
Odds ratios and 95% CI plots for predictors of likelihood of vaccinating people under one’s care (e.g. children)

Risk perceptions are known to be strongly associated with intentions to act to keep oneself healthy and are generally examined along two dimensions: personal susceptibility to the threat and perceptions of severity of the threat [11]. Both were significantly associated with likelihood of vaccinating self (Fig. 1) and vaccinating people under their care including children (Fig. 2). Those who felt that they were more susceptible to COVID-19 and that the consequences are likely to be severe were more willing to vaccinate themselves and people under their care. However, contrary to expectations, someone in the family having COVID-19 was not associated with likelihood of vaccinating self and people under their care including children.

### Major source of COVID-19 news and likelihood of vaccination

News media are major source of information on health and it is more so in the context of COVID-19 given how new the pathogen is. The fragmentation of mass audience [12], means that there are many choices for news and these platforms have distinct editorial slants on the nature and threat of the Pandemic as well as effectiveness of public health mitigation measures. As our data show, the likelihood of getting a COVID-19 vaccine for self and people in their care such as children depends on what groups of platforms serves as “major” source of COVID-19 news. People who relied on mainstream print outlets or newspapers such as the *New York Times* or the *Washington Post* or the *Wall Street Journal* as a major source of COVID-19 news were more likely to vaccinate self and children compared to those who did not rely on such sources. Similarly, people who relied on online media news aggregators such as Google and Yahoo news were more likely to vaccinate self and children compared to those who did not rely on such sources (Fig. 1). In contrast, people who relied on more “conservative” news outlets such as Fox news were less likely to vaccinate themselves or people in their care compared to those who did not rely on such sources. Although social media have received considerable attention both as a key source of health information as well as a platform for anti-vaccine views, our data did not show any significant effects of using social media as a “major source” of COVID-19 news on likelihood of getting the vaccines for self or children (Figs. 1 and 2).

### Party identity, confidence in scientists

To an unusual degree, COVID-19 became a partisan issue with leading political figures with specific political persuasion questioning the seriousness of the threat, public health mitigation measures as well as the credentials of experts. The partisan split appears to be reflected in COVID-19 vaccine acceptance. People who identify themselves as Republicans are less likely to vaccinate self (Fig. 1) or people under their care including children compared to Independents (Fig. 2).

Similarly, those with a great deal of confidence in scientists and researchers are more likely to vaccinate self or children compared to those with “hardly any confidence.” The difference between the two groups is substantial; those with a great deal of confidence in scientists were more than three times more likely to vaccinate self and people in their care including children (Figs. 1 and 2).

### Social determinants

COVID-19 has affected underserved groups –those from lower socioeconomic status, and racial and ethnic minority groups –disproportionately compared to other groups. Communities with larger proportions of minority and immigrant populations and blue-collar workers or low wage earners bore the brunt of job losses, lack of access to health care services and information [13].

The data in our study show a mixed picture. Those with higher education (schooling) were relatively more likely to vaccinate people in their care such as children compared to those with lower schooling.

**Fig. 3:**
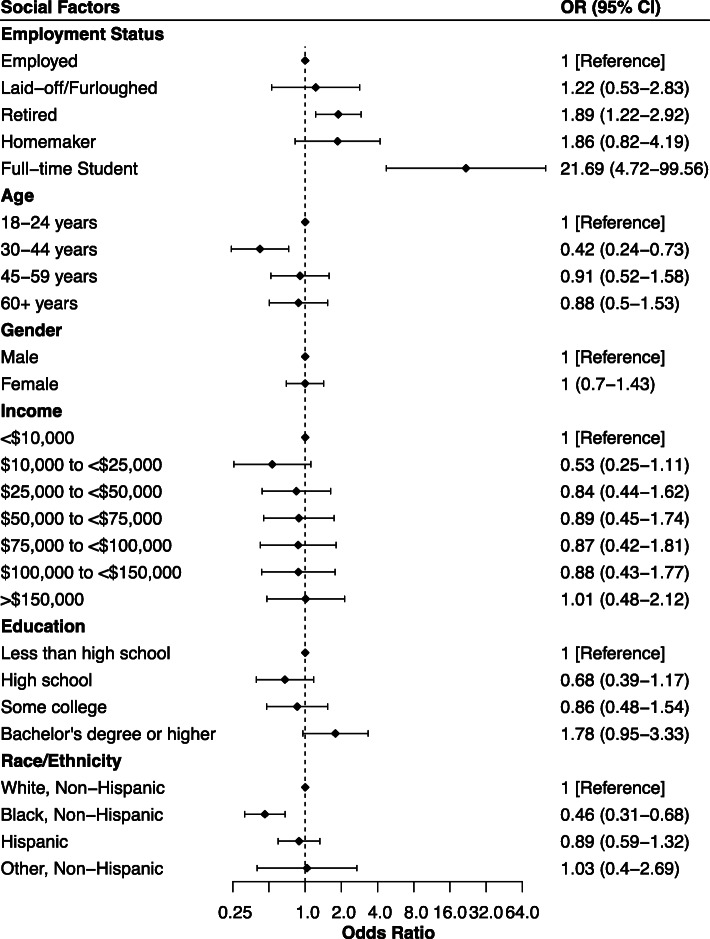
Odds ratios and 95% CI plots for social factors associated with likelihood of vaccinating self

**Fig. 4:**
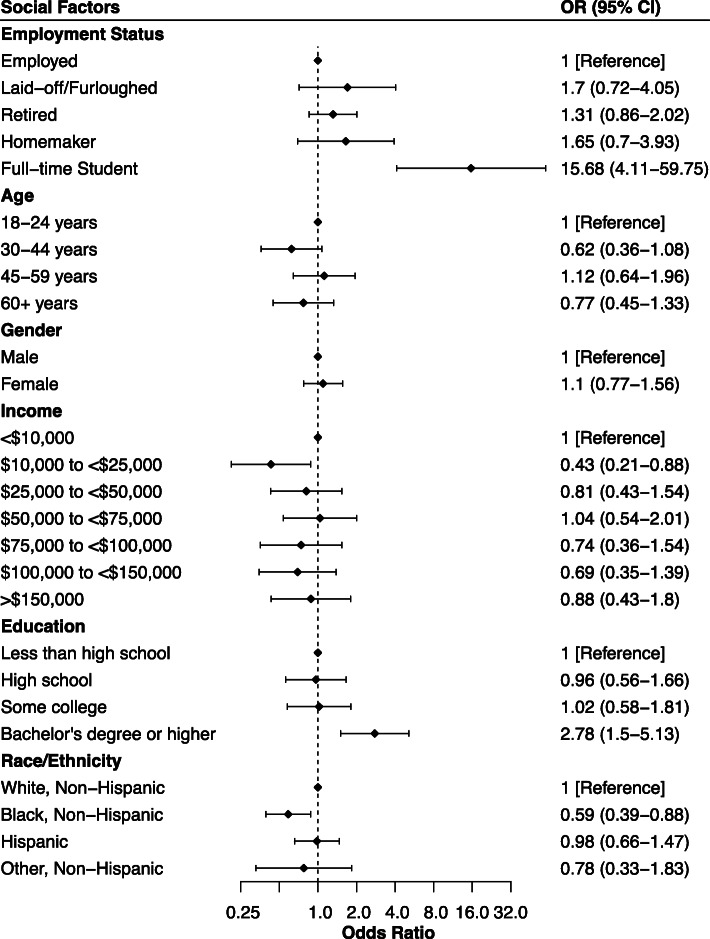
Odds ratios and 95% CI plots for social factors associated with likelihood of vaccinating people under one’s care (e.g. Children)

Among racial and ethnic groups, non-Hispanic Blacks are least likely to agree to vaccinate self (Fig. 3) or people in their care (Fig. 4).

The data also showed that those who are not working are more likely to get vaccines for themselves and people in their care compared to those who are working. When we examined the data further the likelihood of vaccinating was higher among retired and student groups (Figs. 3 and 4).

## Discussion

The widespread global devastation of COVID-19 that has impacted virtually every aspect of life as we know it has engendered various public health and social policies that have received mixed reception. The world has been eager to develop effective treatments and prevention measures such as a vaccine. Several billions of dollars have been committed by governments and foundations to develop the vaccine and the speed with which the development has accelerated is impressive. Now that we have several effective vaccines, the next urgent step is to get people vaccinated to reach herd immunity. A lot of attention and discussion is currently revolving around how to encourage people to get vaccinated especially given the controversies associated with COVID-19 in particular as well as challenges with confidence in vaccines in general. The small but persistent finding that certain proportion of people across the globe are continuing to hesitate to get vaccinated provides a compelling and urgent rationale to examine the factors that drive intentions to vaccinate and vaccination itself. We examined the association between willingness to get a COVID-19 vaccine and those key factors that are known to be associated with health behaviors and vaccination, drawing from a representative national survey of American adults.

Despite the profound and broad-ranging impact of the pandemic the fact that just about 65–68% of the sample is willing to get a vaccine for themselves or children in their charge should be of some alarm. These numbers have improved somewhat in the United States but the vaccine hesitancy among certain sections still remains. The World Health Organization (WHO) reports that immunization programs save 2–3 million lives every year with vaccines to prevent 20 different diseases such as polio, diphtheria, tetanus, smallpox, pertussis, influenza and measles among others. Despite the strong science, and solid health and public health reasons to vaccinate, the proportion of people questioning vaccines, vaccine hesitancy, is a growing threat [14]. Researchers have been careful in distinguishing those groups that are strongly anti-vaccine and who refuse to vaccinate their children or themselves versus those who have doubts and concerns even if they are complying with the recommendations, the vaccine hesitant. In our paper, our questions and cross-sectional design do not permit us to distinguish between those who are hesitant to get a vaccine from those who refuse though we speculate that most of them may be hesitant given the improving trend in vaccinations or intentions to get vaccinated in the United States.

The reasons for this lack of confidence include questions about side effects, safety, efficacy, and the schedule itself. These reasons could lead to potential clusters of unvaccinated or under-vaccinated groups leading to outbreaks that are preventable.

In addition to challenges with vaccine hesitancy, COVID-19 vaccination efforts will have to confront with the poor track record in influenza vaccination rates. Flu vaccination rates in the US are around 60% for children between 6 months and 17 years whereas it was close to 45% for adults with highest rate among 65+ years older adults.

These data on immunization rates, and the data from our study on COVID-19 vaccine acceptance, raise questions along two different dimensions. Clearly, risk perceptions play a role in vaccination [15–17]. In our study the data show that those who perceive that they are susceptible to COVID-19 or who see it as a serious threat are more likely to get vaccinated or vaccinate people in their care compared to those who do not feel that they are susceptible or that the COVID-19 threat is serious. As we speed up vaccination across the globe, vaccine communication campaigns will have to elevate both susceptibility of everyone and the severity of the threat to ensure higher uptake of potential COVID-19 vaccine. However, such public health messaging will have to accompanied by how a vaccine will address the threat and clear directions on how to get the vaccines, in addition ensuring the availability of vaccines to everyone. Public health messaging should also ensure that any perception of unwanted risks, that is safety and efficacy of the vaccines are communicated to groups to reduce hesitancy and counteract anti-vaccine communications are prevalent in social media. It also means drawing on science of public health communication campaigns to ensure the effectiveness of vaccination messages and counter anti-vaccine propaganda.

Studies have also shown that trust in information sources is critical to vaccination intentions and behaviors [14]. Trust in government, medical authorities and one’s own healthcare providers all influence vaccine acceptance. A recent study from Italy on COVID-19 vaccine reported that trust or mistrust in biomedical research is associated with vaccine acceptance [18]. Trust in the potential of COVID-19 vaccine is particularly vulnerable to the political maelstrom that has engulfed almost every action associated with preventing the spread of the infection raising the potential for lower vaccine acceptance. Specifically, political ideology measured through party identification and use of conservative media are strongly associated with unwillingness to get COVID-19 vaccine and this finding has been supported in other studies which show that ideology is associated with believing in anti-vaccine conspiracies [19]. The partisan split seen here in getting the COVID-19 vaccine is complicated by reliance on social media. Several studies and think tanks have repeatedly documented wide-spread anti-vaccine conspiracies and narratives in the social media [19, 20]. The challenge for public health messaging in such an environment is not simple given the “wild west” nature of social media despite recent attempts by social media platforms to flag anti-vaccine content [21, 22]. One potential pathway is to increase trust in science and scientists and communicate the standards and regulatory process for vaccine approvals. Yet a typical establishment approach based on science and facts is unlikely to have a direct influence given that anti-vaccine sentiments are influenced strongly by emotions, often when preferences are driven by “affect heuristic” [23]. It is critical to develop strategies to increase trust while countering the anti-vaccine influences drawing from strategic communication principles.

A series of recent studies have documented the unequal impact of COVID-19-related impact on morbidity and mortality on “vulnerable” groups and communities including people of color, immigrants, those in low-wage occupations and lower incomes. Almost without exceptions almost all these studies show that the social, economic, and health burden is being faced by such groups [24–26]. The fact that there are education or schooling based variations in the likelihood of getting vaccines warrants a more directed and strategic approach. We need to understand the reasons for reluctance among people with low schooling and how to address them. In addition, studies have documented inequalities in communication that deter access to, processing of and capacity to act on information among different social classes which need to be addressed in the context of COVID-19 [27, 28]. In what appears to be counter-intuitive, people who are working or employed are more likely to be reluctant to get a vaccine compared to retired and student populations. While the issue needs to be explored further, the immediate public health implication is targeting this population in public health communications.

Last, the fact that non-Hispanic Blacks were least likely to get a COVID-19 vaccine in our study is not surprising given the history of mistrust among blacks going back to the notorious Tuskegee study but also experiences with the healthcare system and structural racism [29, 30].

## Conclusions

The speed with which COVID-19 spread across the globe and the impact it is having is now a matter of record. Among measures to address the devastating impact is the availability of vaccine to stem the pandemic. While the developments of the vaccines have moved at “warp speed,” the data from this representative national survey show that mere deployment is necessary but not sufficient for vaccines to be accepted by different groups. We need to deploy a pro-active communication campaign strategy to ensure that people are willing to take it and that vaccination is uniform across different population groups.

## Data Availability

The datasets during and/or analyzed during the current study available from the corresponding author on reasonable request.
